# Physical activity trajectory in the first 10 months of the COVID-19 pandemic in Southern Brazil: a follow-up study

**DOI:** 10.1186/s13102-022-00450-0

**Published:** 2022-04-04

**Authors:** Eduardo L. Caputo, Natan Feter, Jayne S. Leite, Igor R. Doring, Júlia Cassuriaga, Felipe M. Delpino, Caroline M. Huckembeck, Ricardo Alt, Marcelo C. da Silva, Airton J. Rombaldi, Felipe F. Reichert

**Affiliations:** 1 Postgraduate Program of Physical Education, Federal University of Pelotas, Pelotas, Brazil; 2 Postgraduate Program of Cardiology, Federal University of Rio Grande do Sul, Porto Alegre, Brazil; 3 Postgraduate Program of Epidemiology, Federal University of Pelotas, Pelotas, Brazil; 4 Study Group of Physical Activity Epidemiology, Federal University of Pelotas, Pelotas, Brazil; 5 Postgraduate Program of Nursing, Federal University of Pelotas, Pelotas, Brazil; 6 School of Physical Education, Federal University of Pelotas, Luís de Camões Street, 625, Pelotas, RS 96055-630 Brazil

**Keywords:** Physical activity, Exercise, Social distancing, Covid-19

## Abstract

**Background:**

A continuous tracking of the PA level during the COVID-19 pandemic is important to understand how people’s behaviour has varied along time. The aim of this study was to evaluate the physical activity (PA) trajectory over the first 10 months of the COVID-19 pandemic in the south of Brazil.

**Methods:**

Data from three timepoints of the PAMPA Cohort were used, as follows: (1) pre-COVID-19 (retrospective); (2) Jun/Jul 2020; (3) Dec 2020/Jan 2021. Self-reported PA practice, frequency, duration, as well as place where activities were performed (at or out of home) were assessed.

**Results:**

A reduction in any (from 68.7 to 47.7%), sufficient (from 41.5 to 22.1%) and out of home PA (from 59.4 to 30.1%) was observed from the first (pre-COVID-19) to the second (Jun/Jul 2020) timepoint, followed by an increase in the third timepoint (Dec 2020/Jan 2021) (60.1%, 37.9%, and 54.3% for any, sufficient, and out of home PA, respectively). The PA trajectory was similar, regardless of sex, educational level or income. Only any (*p* = 0.0007) and sufficient (*p* = 0.0012) PA showed significant interaction with time by sex. Female participants were less likely to engage in any (OR 0.45 95% CI 0.26; 0.77) and sufficient PA (OR 0.40 95% CI 0.24; 0.66).

**Conclusion:**

During the first 10 months of COVID-19 pandemic there was a marked fluctuation on PA pattern in adults from southern Brazil. An ongoing tracking of PA behaviour during COVID-19 pandemic is important to understand how this behaviour varies. Public policies should focus on increasing PA in a higher standard than pre-COVID levels.

**Supplementary Information:**

The online version contains supplementary material available at 10.1186/s13102-022-00450-0.

## Background

The COVID-19 pandemic has caused a profound social, economic, and behavioural impact on worldwide society. Although it is important to keep social distancing, in order to avoid the virus spread, such action might have some deleterious health effects, such as impaired mental health and chronic disease management, as well as problems regarding healthcare access [1–3].

In Brazil there was no national lockdown so far and social distancing measures were adopted at the states and cities level, with a high variability across regions. Rio Grande do Sul, the southernmost state of the country implemented social restrictions measures such as schools’ closure and suspension of events (e.g. music concerts) by March 19th, 2020 [4]. Since then, the level of restrictions varied by all state cities.

Regular physical activity (PA) has positive effects in the treatment and prevention of chronic diseases as well as some infection conditions (i.e. Upper respiratory tract infections) [5, 6]. Due to social restrictions applied to control virus spread, several exercise facilities were closed and people either looked for alternative ways to keep practicing their activities or became sedentary [7, 8]. Regular PA has been associated with a lower risk of outcomes [9], and the severe form of COVID-19 [10], and consequently the risk of hospitalization [11, 12]. To help people keep active during the pandemic, the World Health Organization (WHO) released guidelines and examples of exercises that could be performed at home [13].

PA decreased significantly in the first months of pandemic, especially in women, middle-aged and people with chronic disease [14]. This scenario was also associated with the inequalities related to social restrictions, since only part of the population could remain with their activities during this time [15]. Even though there is consistent evidence showing a reduction in PA when the pandemic started [8], continuous tracking is important to understand how people’s behaviour has varied along time. In addition, PA might be included in gender and economic burden widened by the pandemic [16, 17]. Thus, this study aimed to evaluate the PA trajectory in the first 10 months of the COVID-19 pandemic. We also checked for the PA trajectory according to sex, income and educational level.

## Methods

The present study analyses the first and second wave data from the PAMPA Cohort (Prospective Study About Mental and Physical Health), an ongoing ambispective cohort with adults aged 18 years or more living in the Rio Grande do Sul state, southern Brazil. The first data collection occurred from June 22nd to July 23rd, 2020, which was around three months after the implementation of the first social distancing measures at the state-level, and the second data collection took place on December/2020-January/2021. Brazil registered 2,287,475 cases and 84,082 deaths by the end of the first data collection (July 23rd), and 8,390,341cases and 208,133 deaths by the second (January 15th) [18].

In the first data collection, participants answered questions regarding their current and pre-pandemic PA levels, and in the second data collection participants were again questioned about their current PA levels. Thus, our data have three time points: (1) pre-COVID-19, (2) Jun/Jul 2020, and (3) Dec/2020-Jan/2021.

This study was approved by the local Ethics and Research Committee. The entire protocol might be accessed elsewhere for more details about procedures [19]. All data collection was performed throughout an online-based questionnaire. The first wave was structured via Google Forms, and the second wave via the REDCap platform [20, 21].

Participants were asked to give contact information (i.e., social network name, telephone number) in the first wave. Based on this, all participants who provided contact information were reached to participate again, in the second wave. Participants from all macro-regions (Sul, Centro-Oeste, Missioneira, Vales, Norte, Metropolitana, Serra, in Portuguese) of Rio Grande do Sul state were contacted by social networks (i.e. Facebook, Instagram) or telephone call.

### Physical activity

Participants were asked about their regular PA practice before and during the social distancing period (yes or no), through the following question: “*Were you engaged in physical activity on a regular basis (before/during social distancing/in the last seven days)?*” [22]. For PA practice, those who answered “yes” were asked about the frequency *(“How many days a week do you practice these activities?”)* and duration *(“On the days that you practice these activities, how many minutes on average do they last?”*) of activity practiced. A reliable test–retest correlation was observed when accessing PA with a single item question during the last week [22]. However, this question was adapted aiming the assessment of PA before/during social distancing. Participants were instructed to consider only activities performed on leisure time. For analysis purposes two PA variables were built: (1) any PA, based on the PA practice question (yes or no); and (2) sufficient PA, based on WHO 150 min/week recommendation, where participants were categorized into active or inactive [14, 23].

Participants also indicated where the activities took place (at or out of home). Those activities performed within participant’s household were defined as at home PA, and those performed external to participants’ household (e.g. parks, shared gyms) were defined as out of home PA. The activities performed at home were indicated by the participants: walking/running, rope jumping, stationary bike, strength exercises, stretching exercises, stair climbing, functional training, or any other not quoted before. In addition to the options mentioned for at home activities, the following response options were available for activities performed out of home: aerobics/spinning/step/jump, water exercises, stair climbing, swimming, soccer, volleyball, basketball, tennis, paddle tennis, or other racquet sports, martial arts, and fighting, dance or any other not quoted before. Participants could indicate as many activities they performed. For analysis purposes, only activities with at least 5% prevalence on pre-COVID-19 timepoint were included in the activities list in the results section.

### Covariates

Sex (female, male) and educational level (categorized as high school or lower, university degree, and specialized/Master/Ph.D) were used as covariates. Moreover, to verify the pandemic impact on income, participants were asked if their monthly income had been affected by social distancing measures. The possible answers were “no”, “yes (for less)”, “yes (for more)”. For analysis purposes this variable was dichotomized in yes (reduced) or no.

### Statistical analysis

Data are presented as mean ± 95% Confidence Interval (CI) for numerical, and proportions and 95% CI for categorical variables. The generalized estimating equation (GEE) was used to investigate changes in PA along with the three-time points and how they varied by gender, educational level and income. GEE uses a correlation structure determined a priori, to correct non-independent repeated measures data. To check for PA trajectory by sex, income and educational level an exchangeable correlation structure was used. Results were expressed as predictive margins with the prevalence of PA (any, sufficient and place where PA was performed) and their respective 95% CI for participants who had information regarding PA in the three-time points assessed (n = 675). All analyses were conducted in Stata statistical software (version 15.0; StataCorp LLC, College Station, TX).

## Results

Pre- and during social distancing PA patterns are displayed in Table 1. From the first (pre-COVID-19) to the second (Jun/Jul 2020) timepoint a reduction in any (from 68.7 to 47.7%), sufficient (from 41.5 to 22.1%) and out of home PA (from 59.4 to 30.1%) prevalence, as well as mean PA days (from 2.8 to 1.8) and minutes (38.7 to 25.2) were observed. However, an increase was observed in the third timepoint (Dec 2020/Jan 2021) for any, sufficient, out of home, days and minutes of PA, respectively. Even though these PA variables augmented from the second to the third timepoint, it was not enough to reach the pre-COVID-19 values. Regarding the PA practiced at home, there was no marked differences in the prevalence among timepoints.

**Table 1:** PA patterns across the three timepoints assessed. Rio Grande do Sul, Brazil (n = 675)

	Pre-COVID-19	Jun/Jul 2020	Dec 2020/Jan 2021
*Any PA* (%; 95% CI)	68.7 (63.8; 73.2)	47.1 (42.2; 52.0)	60.1 (55.2; 64.8)
*Sufficient PA* (≥ 150 min/w) (%; 95% CI)	41.5 (36.7; 46.4)	22.1 (18.4; 26.3)	37.9 (33.3; 42.8)
*PA at home* (%; 95% CI)	40.8 (36.1; 45.7)	42.2 (37.4; 47.1)	40.1 (35.4; 45.0)
*PA out of home* (%; 95% CI)	59.4 (54.5; 64.2)	30.1 (25.8; 34.7)	54.3 (49.4; 59.2)
*Days of PA* (mean; 95% CI)	2.8 (2.6; 3.0)	1.8 (1.7; 2.0)	2.4 (2.2; 2.6)
*Minutes of PA* (mean; 95% CI)	38.7 (36.2; 41.1)	25.2 (23.0; 27.5)	35.5 (32.8; 38.3)

Table 2 shows the prevalence of PA types performed at and out of home in the three-timepoints assessed. An increase in most activities performed at home was observed from the first (pre-COVID-19) to the second (Jun/Jul 2020) timepoint (walking/running, strength exercises, rope jumping and flexibility). On the other hand, a reduction in these activities was observed in the third timepoint (Dec 2020/Jan 2021). Regarding activities performed out of home, the prevalence of PA decreased from the first to the second in all activities, and increased in the third timepoint. Even though an increase in activities performed out of home was observed from the second to the third timepoint, it was still lower than pre-COVID-19 levels.

**Table 2:** PA practiced at out of home and in the three timepoints assessed (%; 95% CI). Rio Grande do Sul, Brazil (n = 675)

At home	Pre-COVID-19	Jun/Jul 2020	Dec 2020/Jan 2021
Walking/running	10.6 (7.9; 14.1)	13.5 (10.5; 17.1)	11.4 (8.7; 14.9)
Jump Rope	5.9 (4.0; 8.6)	9.9 (7.3; 13.1)	3.8 (2.3; 6.2)
Stationary bike	5.1 (3.3; 7.7)	4.4 (2.8; 6.8)	5.3 (3.5; 7.9)
Strength exercises	16.8 (13.4; 20.8)	30.2 (25.9; 34.8)	16.5 (13.3; 20.4)
Flexibility exercises	15.8 (12.6; 19.5)	21.8 (18.2; 25.9)	13.3 (10.4; 16.7)
Climb Stairs	13.5 (10.7; 16.8)	11.8 (8.9; 15.4)	11.5 (8.7; 15.0)
*Out of home*			
Walking/running	48.3 (43.4; 53.2)	22.1 (18.3; 26.4)	38.2 (33.5; 43.1)
Jump Rope	5.1 (3.4; 7.8)	2.8 (1.5; 5.1)	3.4 (1.9; 5.9)
Ride bike	21.8 (18.1; 26.1)	10.2 (7.7; 13.5)	13.8 (10.9; 17.3)
Strength exercises	20.5 (16.9; 24.7)	9.9 (7.3; 13.4)	18.2 (14.8; 22.3)
Flexibility exercises	12.2 (9.5; 15.5)	5.9 (3.9; 8.7)	10.2 (7.6; 13.7)
Climb Stairs	8.9 (6.5; 12.1)	3.9 (2.5; 6.4)	2.6 (1.3; 5.2)

**Fig. 1:**
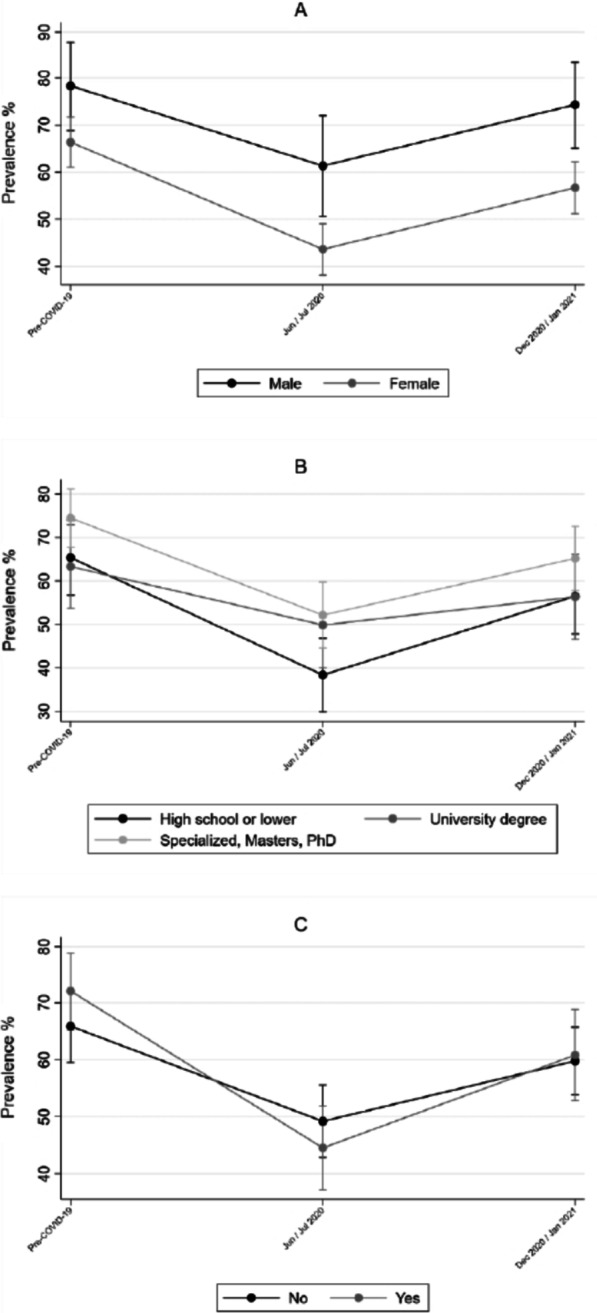
Prevalence of any PA before and during COVID-19 pandemic for sex (**A**), educational level (**B**) and decreased monthly income (**C**). Rio Grande do Sul, Brazil. (n = 675)

**Fig. 2:**
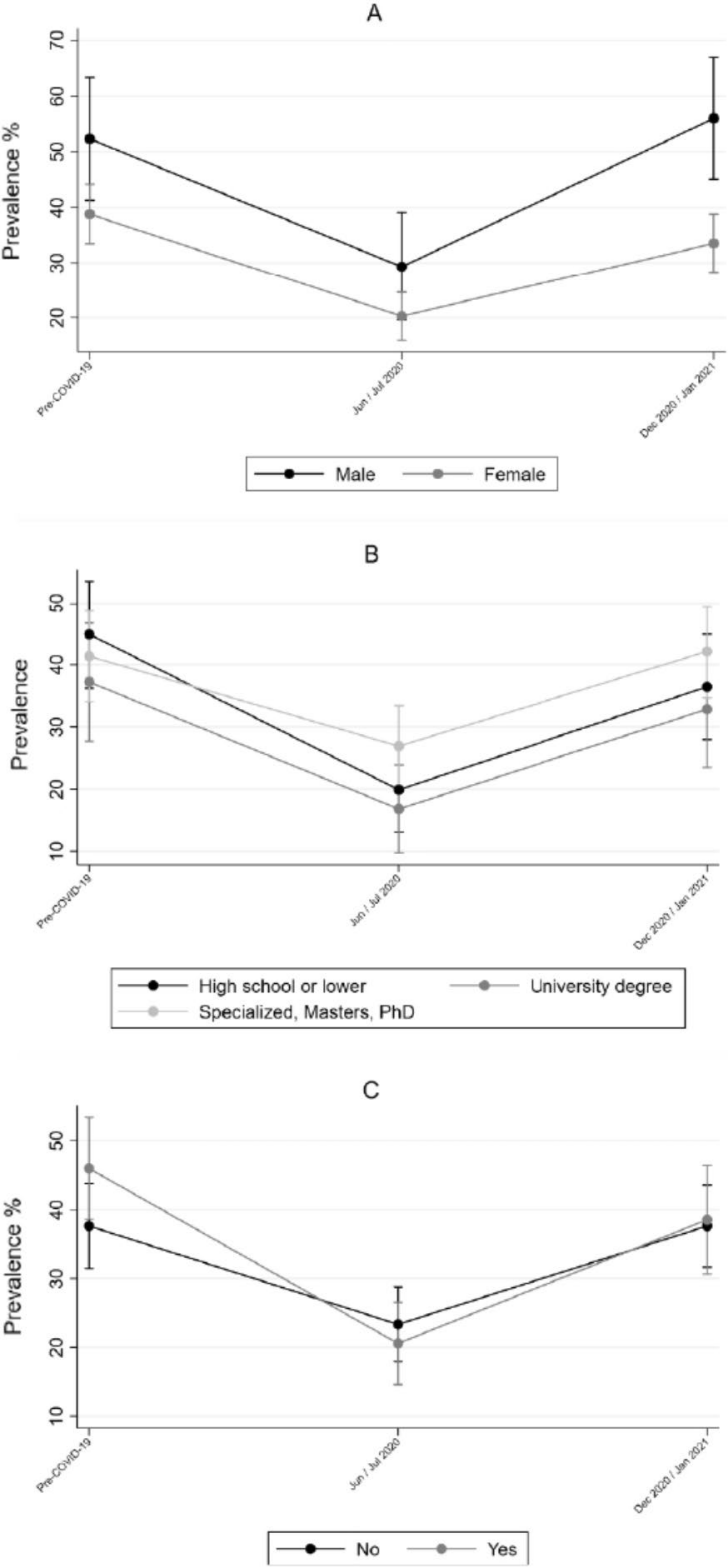
Prevalence of sufficient PA before and during COVID-19 pandemic for sex (**A**), educational level (**B**) and decreased monthly income (**C**). Rio Grande do Sul, Brazil. (n = 675)

**Fig. 3:**
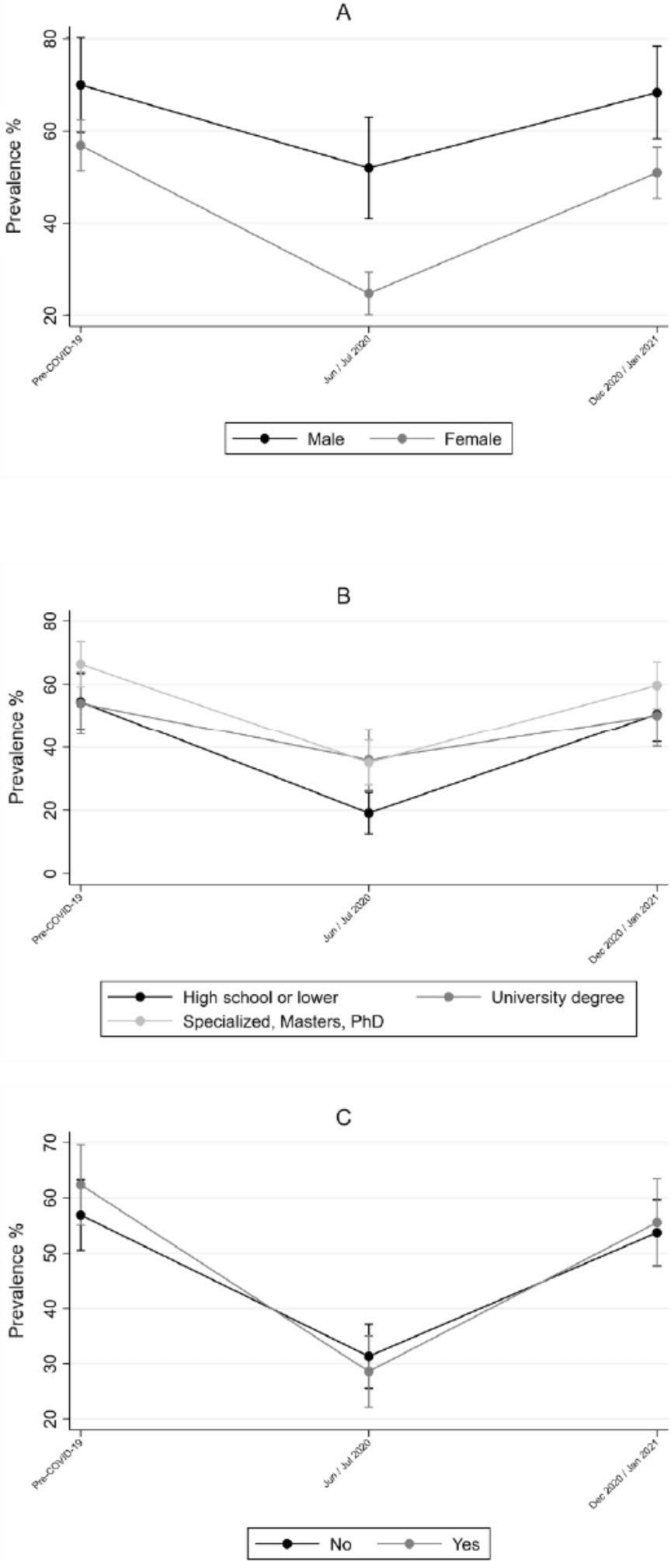
Prevalence of PA performed out of home before and during COVID-19 pandemic for sex (**A**), educational level (**B**) and decreased monthly income (**C**). (n = 675)

PA over the three timepoints by sex, educational level and income are displayed in Figs. 1, 2 and 3. Descriptively, any, sufficient, and out of home PA decreased in the second and increased in the third timepoint, regardless of sex, educational level or income. A distinct trajectory was observed in PA performed at home (Additional file 1). A significant interaction with time was observed only by sex on any (*p* = 0.0012) and sufficient PA (*p* = 0.0007), where female participants were less likely to engage in PA (OR 0.45 95% CI 0.26; 0.77, and OR 0.40 95% CI 0.24; 0.66, for any and sufficient PA, respectively) (Additional file 2).

## Discussion

Our study revealed a marked fluctuation in PA patterns during the COVID-19 pandemic. After a reduction in the first months of pandemic, an increase in PA levels was observed in roughly 10 months of social distancing, yet it did not reach the same level as before the pandemic. Also, women showed decreased PA when compared to men. This PA scenario during social distancing was evident regardless of socioeconomic characteristics, such as educational and income level.

The decrease in PA levels in the first months of the COVID-19 pandemic was expected due to social restrictions imposed by governments to reduce virus spread [8]. By this time (Jun/Jul 2020) Brazil reported an increase of 25% in cases rate [24], and there was little knowledge on how long the pandemic would last, what social effects it would have, and how people would react to government measures. From May 2020 to May 2021, the Rio Grande do Sul state adopted the “Controlled Social Distancing Model”, which outlined the risk of COVID-19 infection in a flag color-coded approach. Each region was evaluated by several health indicators (e.g. ICU availability, rate of new COVID-19 cases), and regions of the state were categorized based on risk of infection, as follows: low (yellow), medium (orange), high (red), and extremely high (black) [25]. In the first data collection (Jun/Jul 2020), all the Rio Grande do Sul state was either at orange or red colours, which indicated a strengthened social restriction level, corresponding with the lower PA level observed. Such levels could also be related to the fact that the winter (Jun/Jul/Aug) in Rio Grande do Sul is very cold and humid, which is a barrier to PA [26]. Similarly, by Dec/Jan, Brazil reported a 40% increase in cases rate, and the social restrictions were strengthened after a period of easiness. Thus, we hypothesized that PA levels would be similar to Jun/Jul assessment. Nevertheless, the increase in PA observed in Dec/Jan might be related to weather conditions (summer season), since many people feel encouraged to practice PA, especially outdoors. In addition, several individuals did not follow government’s recommended social distancing instructions, which might also explain our findings [27]. A survey conducted in Brazil indicated that lack of appropriate facilities and equipment, were barriers to PA practice during pandemic [28]. In both cohort assessments, gyms and sports facilities were closed in most cities of the Rio Grande do Sul state. Thus, people had to find other ways and spaces to remain active. A decreased PA in the first assessment (Jun/Jul) of social restrictions followed by an increase in the second assessment (Dec/Jan) was observed for activities performed out of home. This was expected, since summer season (Dec/Jan/Feb) is tempting to perform PA outdoors, and people were never forbidden of walking or riding bicycles as well as performing exercises in public spaces (e.g. parks). The weak social restrictions adopted by the state, along with encouraging messages from the national government saying that people could keep their “normal lives” and routines despite the pandemic [27], could also be related to the increased practice of PA out of home.

The PA health-related benefits are not directly associated with its practice in sports facilities or gym clubs. Home-based exercises and workouts can also bring benefits to health [29, 30], and this should be emphasized during pandemic times, such as the COVID-19, when social distancing is required. Furthermore, public policies are necessary to promote a healthy lifestyle and increase PA habits regardless of settings (i.e. home), to avoid external influences, such as weather, for example.

The gender inequalities related to PA are well described [31] and were further aggravated because of COVID-19 pandemic social restrictions. The burden on women regarding home chores and care taking was even more evident with the routine imposed by the pandemic, as well as the social barriers related to PA practice for this group [15]. These inequalities have a negative effect on women’s leisure time, which is related to their lack of time and possibility to include PA in their daily routine [15]. Also, due to these inequalities, it is possible that women are more likely to experience incidental PA, such as home-related chores [32]. Unfortunately, we have only assessed leisure-time PA.

Educational level is a strong determinant of leisure-time PA. People with high educational levels are more likely to have access and resources to engage in healthy behaviours, such as PA [33]. There were no marked differences of PA prevalence among educational level groups, with a difference only in the Jun/Jul timepoint for activities performed out of home, where participants with low educational level (i.e. High school or low) showed less PA. However, our sample has a high proportion of participants with academic degree (40.2%), which is overexpressed when compared to the national data (16.9%) [1, 34]. The high educational level of our sample might hide disparities among classes and explain the small education effect of PA in our results.

### Strength and limitations

Some limitations of our study should be listed. First, our study presented a retention rate of 52% [19], which is not very high. However, even studies in wealthier countries, where more people have internet access achieved similar response rates when compared to our study [35]. Second, as face-to-face interviews were not allowed by ethics boards in Brazil at the time of our data collection, self-reported assessments were used. Even though wearable devices may address any self-report bias concern, they also have their limitations such as wear time and device validity [36, 37]. Third, as previously stated, our sample has a high proportion of participants with an academic degree, thus, selection bias is an issue since less educated people have limited access to internet [38]. Thus, one must be cautions to extrapolate our findings on PA trajectory to the whole population, since PA levels are different across schooling levels of Brazilians. Fourth, PA level of the first timepoint was assessed retrospectively, and recall bias cannot be ruled out. Fifth, since large internet-based survey such as this was not usual among the Brazilian population before the pandemics, there is no evidence that instruments usually applied in face-to-face interviews such as the IPAQ has the same validity when applied online. In addition, our study had an extensive questionnaire which are associated with higher non-response rate. Thus, we opted for a simplified measure of PA [38]. However, one feature of our study stands out, we tracked PA behaviour from pre-COVID-19 levels into 10 months of social restrictions. Such information can contribute to a better understanding of not only how people behave, but also how the pandemic scenario has affected their health on a long term.

### Perspective

COVID-19 pandemic raised the concern with physical inactivity due to social restriction measures. Although several studies were conducted at the beginning of pandemic showing a decrease on PA levels, our study demonstrated how PA fluctuated along the first 10 months of COVID-19 pandemic, and it is still far from desirable levels. Taking these findings into account, an ongoing tracking and assessment of PA behaviour during COVID-19 is important to understand how this behaviour varies and what actions are needed in order to increase PA on populational level.

## Conclusion

PA showed a marked fluctuation during the first 10 months of COVID-19, decreasing in the first months and increasing afterwards. However, the latter increase was not enough to reach the pre-pandemic PA levels. This pattern was observed regarding PA practice, minutes and days of PA per week, as well as activities performed out of home. Public policies should focus on increasing PA to higher standards than pre-COVID levels.

## Supplementary Information

**Additional file 1**. Suppementary Material.

**Additional file 2**. Supplementary Material.

## Abbreviation

PA: Physical activity

## Declarations

### Availability of data and materials

The datasets used and/or analysed during the current study are available from the corresponding author on reasonable request.

## References

[1] Feter N, Caputo EL, Doring IR, Leite JS, Cassuriaga J, Reichert FF (2021). Sharp increase in depression and anxiety among Brazilian adults during the COVID-19 pandemic: findings from the PAMPA cohort. Public Health.

[2] Feter N, Caputo EL, Smith EC, Doring IR, Cassuriaga J, Leite JS, et al. Association between physical activity and subjective memory decline triggered by the COVID-19 pandemic: findings from the PAMPA cohort. Prevent Med. 2021;145. 10.1016/j.ypmed.2020.106415 PMC7833596 33400938

[3] Leite JS, Feter N, Caputo EL, Doring IR, Cassuriaga J, Reichert FF (2021). Managing noncommunicable diseases during the covid-19 pandemic in brazil: findings from the pampa cohort. Ciencia e Saude Coletiva.

[4] Da Silva LLS, Lima AFR, Polli DA, Razia PFS, Pavão LFA, De Hollanda Cavalcanti MAF, et al. Social distancing measures in the fight against covid-19 in brazil: Description and epidemiological analysis by state. Cadernos de Saude Publica. 2020;36. 10.1590/0102-311X00185020 32965378

[5] Nieman DC, Wentz LM (2019). The compelling link between physical activity and the body’s defense system. J Sport Health Sci.

[6] Warburton DER, Bredin SSD (2017). Health benefits of physical activity: A systematic review of current systematic reviews. Curr Opin Cardiol.

[7] Dunton GF, Wang SD, Do B, Courtney J (2020). Early effects of the COVID-19 pandemic on physical activity locations and behaviors in adults living in the United States. Prevent Med Rep.

[8] Caputo EL, Reichert FF. Studies of physical activity and COVID-19 during the pandemic: a scoping review. J Phys Act Health. 2020;17. 10.1123/jpah.2020-0406 33152693

[9] Zhang X, Li X, Sun Z, He Y, Xu W, Campbell H (2020). Physical activity and COVID-19: an observational and Mendelian randomisation study. J Glob Health.

[10] Sallis R, Young DR, Tartof SY, Sallis JF, Sall J, Li Q (2021). Physical inactivity is associated with a higher risk for severe COVID-19 outcomes: a study in 48 440 adult patients. Br J Sports Med.

[11] Filgueira TO, Castoldi A, Santos LER, de Amorim GJ, de Sousa Fernandes MS, Anastácio WDLDN (2021). The relevance of a physical active lifestyle and physical fitness on immune defense: mitigating disease burden, with focus on COVID-19 consequences. Front Immunol.

[12] Hamer M, Kivimäki M, Gale CR, Batty GD (2020). Lifestyle risk factors, inflammatory mechanisms, and COVID-19 hospitalization: a community-based cohort study of 387,109 adults in UK. Brain Behav Immun.

[13] WHO. Stay physically active during self-quarantine. 2020. https://www.euro.who.int/en/health-topics/health-emergencies/coronavirus-covid-19/publications-and-technical-guidance/noncommunicable-diseases/stay-physically-active-during-self-quarantine. Accessed 1 Jun 2021.

[14] Caputo EL, Feter N, Doring IR, Leite JS, Cassuriaga J, Rombaldi AJ (2021). How has COVID-19 social distancing impacted physical activity patterns? Data from the PAMPA cohort, Brazil. J Exerc Sci Fit.

[15] Crochemore-Silva I, Knuth AG, Wendt A, Nunes BP, Hallal PC, Santos LP (2020). Physical activity during the COVID-19 pandemic: a population-based cross-sectional study in a city of south Brazil. Ciencia e Saude Coletiva.

[16] Nordhues HC, Bhagra A, Stroud NN, Vencill JA, Kuhle CL (2021). COVID-19 gender disparities and mitigation recommendations: a narrative review. Mayo Clin Proc.

[17] Suthar S, Das S, Nagpure A, Madhurantakam C, Tiwari SB, Gahlot P (2021). Epidemiology and diagnosis, environmental resources quality and socio-economic perspectives for COVID-19 pandemic. J Environ Manag.

[18] CONASS. PAINEL CONASS | COVID-19. https://www.conass.org.br/painelconasscovid19/. Accessed 6 Jul 2021.

[19] Feter N, Caputo EL, Doring IR, Leite JS, Cassuriaga J, Felipe, et al. Longitudinal study about low back pain, mental health, and access to healthcare system during COVID-19 pandemic: protocol of an ambispective cohort Short title: PAMPA cohort: study protocol. Cold Spring Harbor Laboratory Press; 2020.

[20] Harris PA, Taylor R, Thielke R, Payne J, Gonzalez N, Conde JG (2009). Research electronic data capture (REDCap)—a metadata-driven methodology and workflow process for providing translational research informatics support. J Biomed Inform.

[21] Harris PA, Taylor R, Minor BL, Elliott V, Fernandez M, O’Neal L (2019). The REDCap consortium: building an international community of software platform partners. J Biomed Inform.

[22] Milton K, Bull FC, Bauman A (2011). Reliability and validity testing of a single-item physical activity measure. Br J Sports Med.

[23] WHO. WHO guidelines on physical activity and sedentary behaviour. 2020. https://www.who.int/publications/i/item/9789240015128. Accessed 3 Dec 2020.

[24] Brazil: Coronavirus pandemic country profile-our world in data. https://ourworldindata.org/coronavirus/country/brazil. Accessed 12 Jan 2022.

[25] Rio Grande do Sul State Governament. Rio Grande do Sul Coronavírus Panel. https://coronavirus.rs.gov.br/inicial. Accessed 28 May 2021.

[26] Silva KS, Del Duca GF, Garcia LMT, da Silva JA, Bertuol C, de Oliveira ESA (2016). Barriers associated with frequency of leisure-time physical activity among Brazilian adults of different income strata. Scand J Med Sci Sports.

[27] WHO/Europe discusses how to deal with pandemic fatigue. https://www.who.int/news-room/feature-stories/detail/who-europe-discusses-how-to-deal-with-pandemic-fatigue. Accessed 28 May 2021.

[28] Farah BQ, do Prado WL, Malik N, Lofrano-Prado MC, de Melo PH, Botero JP (2021). Barriers to physical activity during the COVID-19 pandemic in adults: a cross-sectional study. Sport Sci Health.

[29] Chen HM, Tsai CM, Wu YC, Lin KC, Lin CC (2015). Randomised controlled trial on the effectiveness of home-based walking exercise on anxiety, depression and cancer-related symptoms in patients with lung cancer. Br J Cancer.

[30] Peng X, Su Y, Hu Z, Sun X, Li X, Dolansky MA (2018). Home-based telehealth exercise training program in Chinese patients with heart failure: a randomized controlled trial. Medicine.

[31] Dumith SC, Hallal PC, Reis RS, Kohl HW (2011). Worldwide prevalence of physical inactivity and its association with human development index in 76 countries. Prev Med.

[32] Orlandi M, Rosselli M, Pellegrino A, Boddi M, Stefani L, Toncelli L (2021). Gender differences in the impact on physical activity and lifestyle in Italy during the lockdown, due to the pandemic. Nutr Metab Cardiovasc Dis.

[33] Huikari S, Junttila H, Ala-Mursula L, Jämsä T, Korpelainen R, Miettunen J (2021). Leisure-time physical activity is associated with socio-economic status beyond income—cross-sectional survey of the Northern Finland Birth Cohort 1966 study. Econ Hum Biol.

[34] IBGE. Pesquisa Nacional por Amostras de Domícilios Contínua (PNAD Contínua). Rio de Janeiro; 2019.

[35] Matt Brown A, Goodman A, Peters A, Ploubidis GB, Sanchez A, Silverwood R (2020). COVID-19 survey in five national longitudinal studies waves 1 and 2 user guide (version 2) how to cite this guide survey in five national longitudinal studies: waves 1 and 2 user guide (version 2).

[36] Ding D, Cheng M, del Pozo CB, Lin T, Sun S, Zhang L (2021). How COVID-19 lockdown and reopening affected daily steps: evidence based on 164,630 person-days of prospectively collected data from Shanghai, China. Int J Behav Nutr Phys Act.

[37] Kingsnorth AP, Patience M, Moltchanova E, Esliger DW, Paine NJ, Hobbs M (2021). Changes in device-measured physical activity patterns in U.K. adults related to the first COVID-19 lockdown. J Meas Phys Behav.

[38] Caputo EL, Feter N, Rombaldi AJ, da Silva MC, Reichert FF (2021). What are the challenges of epidemiological research during the COVID-19 pandemic?. Motriz Revista de Educacao Fisica.

