# Supplementary material for: Physical activity trajectory in the first 10 months of the COVID-19 pandemic in Southern Brazil: a follow-up study
Source: BMC Sports Sci Med Rehabil. 2022 Apr 4;14:58. doi: 10.1186/s13102-022-00450-0 (PMC8978170; doi:10.1186/s13102-022-00450-0)
Supplement: Supplementary file 1 — Additional file 1. Suppementary Material. [file 13102_2022_450_MOESM1_ESM.docx]

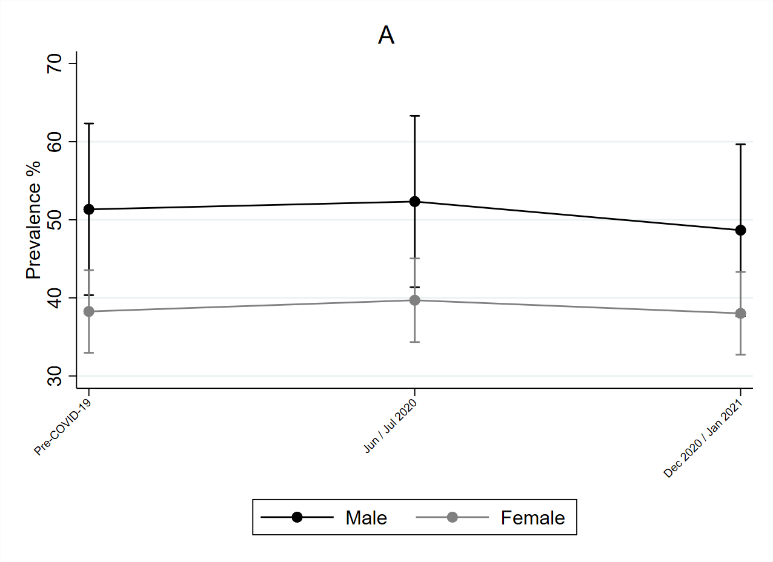


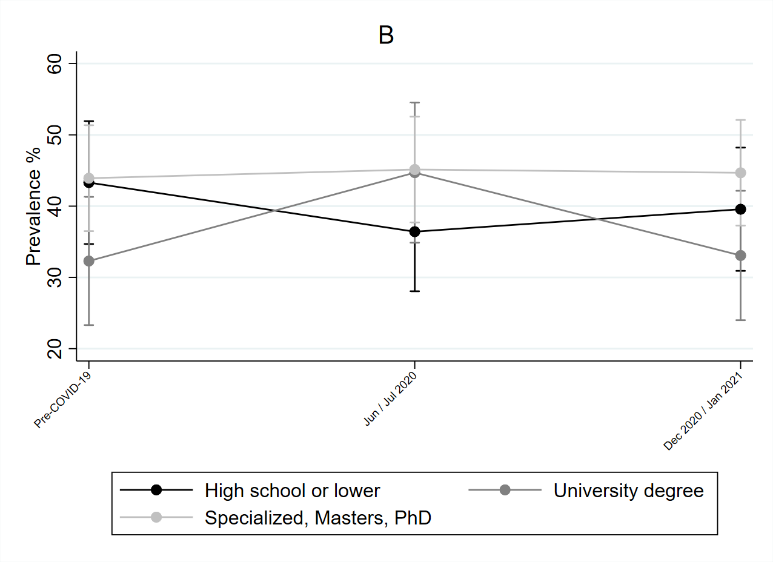


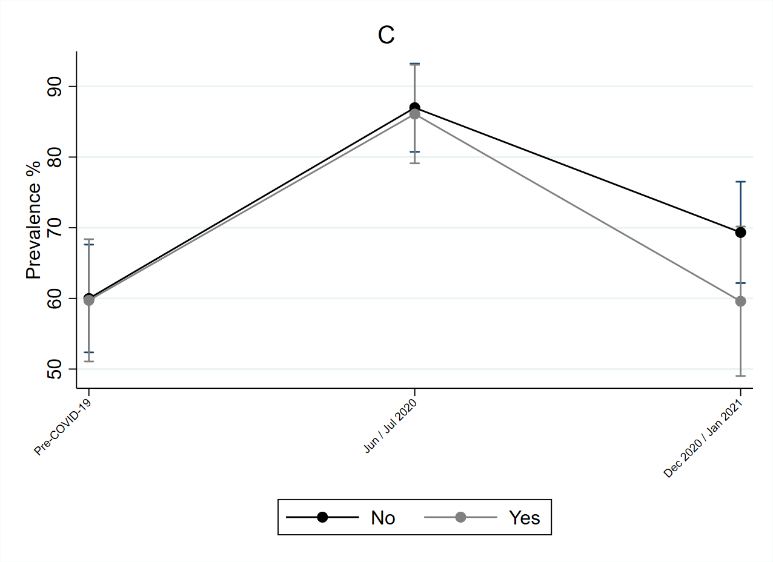


**Supplementary Material**. Prevalence of PA performed at home before and during COVID-19 pandemic for sex (A), educational level (B) and decreased monthly income (C). Rio Grande do Sul, Brazil. (n = 675)
