# Supplementary material for: Physical activity trajectory in the first 10 months of the COVID-19 pandemic in Southern Brazil: a follow-up study
Source: BMC Sports Sci Med Rehabil. 2022 Apr 4;14:58. doi: 10.1186/s13102-022-00450-0 (PMC8978170; doi:10.1186/s13102-022-00450-0)
Supplement: Supplementary file 2 — Additional file 2. Supplementary Material. [file 13102_2022_450_MOESM2_ESM.docx]

| **Supplementary Material2.** PA by sex, educational level and income over time. | | | |
| --- | --- | --- | --- |
|  | **OR (95%CI)** | **p-value** | **Group x Time** |
| ***Any PA*** |  |  |  |
| Sex | 0.45 (0.26; 0.77) | 0.004 | 0.0012 |
| Educational level | 1.20 (0.94; 1.53) | 0.135 | 0.0991 |
| Income | 1.04 (0.70; 1.57) | 0.837 | 0.3531 |
| ***Sufficient PA*** |  |  |  |
| Sex | 0.40 (0.24; 0.66) | <0.001 | 0.0007 |
| Educational level | 1.13 (0.89; 1.43) | 0.325 | 0.1877 |
| Income | 1.04 (0.69; 1.58) | 0.855 | 0.2754 |
| ***PA at home*** |  |  |  |
| Sex | 1.04 (0.56; 1.94) | 0.901 | 0.5744 |
| Educational level | 0.95 (0.69; 1.30) | 0.731 | 0.1509 |
| Income | 0.65 (0.38; 1.13) | 0.127 | 0.5169 |
| ***PA out of home*** |  |  |  |
| Sex | 0.73 (0.25; 2.15) | 0.564 | 0.5601 |
| Educational level | 1.06 (0.66; 1.70) | 0.809 | - |
| Income | 1.50 (0.66; 3.43) | 0.336 | 0.6096 |
